# Oxygen surface exchange kinetics measurement by simultaneous optical transmission relaxation and impedance spectroscopy: Sr(Ti,Fe)O_3-x_ thin film case study

**DOI:** 10.1080/14686996.2018.1430448

**Published:** 2018-02-20

**Authors:** Nicola H. Perry, Jae Jin Kim, Harry L. Tuller

**Affiliations:** ^a^ International Institute for Carbon-Neutral Energy Research (WPI-I^2^CNER), Kyushu University, Fukuoka, Japan; ^b^ Department of Materials Science and Engineering, Massachusetts Institute of Technology, Cambridge, MA, USA

**Keywords:** Thin films, perovskite, mixed ionic and electronic conductor, oxygen surface exchange, optical absorption, impedance spectroscopy, 50 Energy Materials, 107 Glass and ceramic materials, 207 Fuel cells / Batteries / Super capacitors, 206 Energy conversion / transport / storage / recovery; 204 Optics / Optical applications, 212 Surface and interfaces, 306 Thin film / Coatings, 307 Kinetics and energy / mass transport, 201 Electronics / Semiconductor / TCOs, 205 Catalyst / Photocatalyst / Photosynthesis

## Abstract

We compare approaches to measure oxygen surface exchange kinetics, by simultaneous optical transmission relaxation (OTR) and AC-impedance spectroscopy (AC-IS), on the same mixed conducting SrTi_0.65_Fe_0.35_O_3-x_ film. Surface exchange coefficients were evaluated as a function of oxygen activity in the film, controlled by gas partial pressure and/or DC bias applied across the ionically conducting yttria-stabilized zirconia substrate. Changes in measured light transmission through the film over time (relaxations) resulted from optical absorption changes in the film corresponding to changes in its oxygen and oxidized Fe (~Fe^4+^) concentrations; such relaxation profiles were successfully described by the equation for surface exchange-limited kinetics appropriate for the film geometry. The k_chem_ values obtained by OTR were significantly lower than the AC-IS derived k_chem_ values and k_q_ values multiplied by the thermodynamic factor (bulk or thin film), suggesting a possible enhancement in k by the metal current collectors (Pt, Au). Long-term degradation in k_chem_ and k_q_ values obtained by AC-IS was also attributed to deterioration of the porous Pt current collector, while no significant degradation was observed in the optically derived k_chem_ values. The results suggest that, while the current collector might influence measurements by AC-IS, the OTR method offers a continuous, *in situ*, and contact-free method to measure oxygen exchange kinetics at the native surfaces of thin films.

## Graphical abstract

**Figure:**
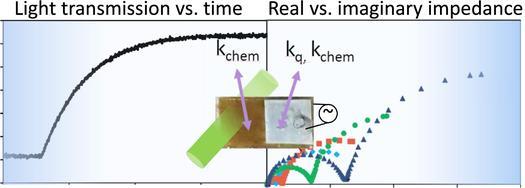

## 1. Introduction

Oxide thin films have been undergoing widespread development for both advancement of device performance and fundamental scientific studies. A number of useful functionalities arise from their well-defined and low-dimensional geometries, compared to their bulk counterparts, including lower (cross-plane) electrical resistance, higher optical transmittance, and surface exchange- rather than bulk diffusion-dominated kinetics. Some of these effects scale with the geometry (trivial size effects) while other nonlinear changes can emerge at the limit of nanoscale and confined thin films (true size effects) [1]. Thin films have therefore enabled a number of novel components and studies: low resistance electrolytes [2] and active functional layers [3] in solid oxide fuel cells (SOFCs), sensitive chemi-resistive gas detectors [4], transparent and/or flexible conducting layers for photovoltaics, electronics, and batteries [5,6], and fundamental insights into SOFC electrode processes using model electrode systems [7], to offer just a few examples. The transport behavior and reactivity enabling these applications depend on the underlying point defect thermodynamics and reaction kinetics in the thin films, and so it is important to be able to measure, understand, and thereby rationally manipulate these properties. Some of the established methods for probing point defect behavior (particularly stoichiometry deviations) in bulk oxides, such as thermogravimetric analysis, iodometric titration, or neutron diffraction (with limited precision), are challenging to apply to thin film samples, given their small volumes, although coulometric titration may be appropriate [8]. Since thin film point defect chemistry can significantly differ from bulk defect chemistry [9] – owing to effects including processing-induced metastability [10], substrate- and/or processing-induced strain [11,12], high interface-to-volume ratios, and interface polarity/ charge-transfer/ space charge effects [13] – development of new thin film point defect evaluation techniques is preferable, rather than reliance upon bulk data. Similarly, when considering measurement of surface exchange kinetics, the established techniques traditionally applied to bulk samples (tracer diffusion, electrical conductivity relaxation) are limited by being either non-continuous, *ex situ*, or dependent on contact between the surfaces under study and metallic current collectors. To truly understand materials performance in devices, one should characterize their kinetics over time to understand stability, in realistic operating conditions, and on native surfaces unaffected by current collectors, which could otherwise enhance or hinder catalytic activity of the specific surfaces being probed.

In this paper we apply a continuous, contact-free, and *in situ* optical transmission relaxation (OTR) approach, which can enable measurement of native oxygen surface exchange kinetics as well as steady-state point defect chemistry over a range of environmental conditions in thin films. Here, we focus on measuring the oxygen surface exchange kinetics of the perovskite SrTi_0.65_Fe_0.35_O_3-x_ (STF35) as a case study. In another publication we investigate in more depth the relationships between steady-state optical absorption, electronic structure, and point defect chemistry in STF thin films [14]. In a third study we apply the OTR method to understand the relationship between microstructure and surface exchange kinetics, as well as their coupled evolution over time, in STF35 films [15,16]. The OTR method has recently been applied to measure kinetics [17] and point defect concentrations [18] in films of the fluorite-structured Ce_0.9_Pr_0.1_O_2-x_, and so the present work contributes to demonstrating wider applicability of this technique on a system with a different crystal, electronic, and point defect structure. Prior to these recent thin film studies, OTR had been developed to study reaction kinetics and solid state diffusion primarily in various bulk or single-crystal materials [19–23]. Additionally, we compare surface exchange kinetics measured by the OTR approach with simultaneously measured kinetics by AC-impedance spectroscopy (AC-IS) on the same sample. We examine whether these varied techniques can provide similar results, thereby providing insight into any differences that may be encountered when applying these approaches.

STF35 is a mixed ionic and electronic conductor with a wide stability range in terms of temperature and oxygen partial pressure [24], of interest for both fuel [25] and oxygen [26] electrodes of solid oxide cells as well as for oxygen sensors [27]. Aside from use of the OTR technique, the oxygen surface exchange kinetics of STF (of various compositions) have previously been studied by isotope exchange depth profiling [28] and electrical conductivity relaxation [29] on bulk samples, pulse-response isotope exchange [30] on powder samples, and AC impedance spectroscopy on thin films [31], demonstrating rapid exchange kinetics that are comparable to those of known high-performance electrode materials [32], degradation over time correlating to Sr surface segregation [33], a strong dependence of kinetics on crystallinity [15], and a possible rate-limiting role of electron transfer as demonstrated by sensitivity of the surface exchange activation enthalpy to bulk Fermi level [32]. As STF has been so widely studied, it represents an ideal composition to test by the OTR approach, to probe this newer technique’s wider utility, benefits, and drawbacks. In the present work we apply OTR to measure the oxygen surface exchange kinetics as a function of oxygen partial pressure and time on transparent substrates at 600 °C. We also compare the optical results (*k*_chem_) with simultaneously measured kinetics by AC impedance spectroscopy (*k*_chem_ and *k*_*q*_), using estimated bulk and thin film thermodynamic factors for relating *k*_chem_ and *k*_*q*_, to infer the possible influence of the current collector in the latter technique. Thin film thermodynamic factors, estimated from the measured film capacitances, were higher than the bulk values and dependent on the current collector material, suggesting a possible interfacial contribution. Owing to this potential distortion of the true film thermodynamic factors, bulk thermodynamic factors were also applied in the analysis. Comparison of the OTR and AC-IS results demonstrates a significant difference in the surface exchange coefficients measured by the two approaches, with the faster kinetics measured by AC-IS, possibly owing to the Pt or Au current collectors. Additionally, over prolonged measurement time, the kinetics as measured by AC-IS demonstrated a significant deterioration, while those measured optically did not. Based on analysis of the other impedance features for the cell with the porous Pt current collector and counter electrode (ohmic offset, Pt counter electrode), the decrease in apparent STF *k*_chem_ and *k*_*q*_ over time was attributed primarily to degradation of the Pt current collector.

## 2. Experimental approach

A ~200 nm thick STF35 film was grown by pulsed laser deposition (PLD) from a highly dense ceramic target, onto a (1 0 0)-oriented, two-sides polished 10 × 10 × 0.5 mm, 13 mol% Y_2_O_3_ – stabilized ZrO_2_ (YSZ) single-crystal substrate (Dalian Keri Optoelectronic Technology Co. Ltd., Dalian, China) at 700 °C. Details of target preparation and PLD conditions are given elsewhere [34,35]. Growth rates and film thicknesses were calibrated using X-ray reflectivity measurements of films grown for short times (~10 nm thick) and cross-sectional scanning electron microscopy images of thicker films (~few hundred nm thick). Out-of-plane crystallographic orientation and crystalline quality were evaluated by X-ray diffraction 2*θ*-*ω* (coupled) scans and ω (rocking curve) scans, respectively. The film exhibited a (1 1 0) out-of-plane orientation and excellent crystalline quality (rocking curve full width at half maximum value ~ 0.05°), as described in our earlier work [34], with columnar grains having lateral dimensions of ~20–50 nm (see Ref. [15] for further microstructural analysis of similarly-fabricated STF films). The sample was cut in half prior to measurements to fit into the small tube (~1 cm diameter) for OTR measurements.

The OTR approach was applied to measure oxygen surface exchange kinetics by probing the change over time in monochromated and collimated light intensity transmitted through the film as it oxidized or reduced in response to a rapid, small change in the oxygen partial pressure in the surrounding gas atmosphere. The technique is analogous to the established electrical conductivity relaxation technique [36,37], which relies on changes in electrical conductivity over time being proportional to changes in oxygen content in the sample. For example, for mixed conductors with p-type behavior and sufficiently high electronic transference numbers (e.g. in oxidizing conditions – high pO_2_ and low temperature), the change in measured conductivity upon changing pO_2_ corresponds to a change in hole concentration, if one assumes a constant hole mobility vs. pO_2_, and the change in hole concentration can be related linearly to a change in oxygen content by an electroneutrality condition. In the OTR approach being examined here, a change in oxygen content additionally results in a linear change in the optical absorption coefficient of the sample at a wavelength characteristic of a defect derived color center. This Beer–Lambert law relationship, i.e. proportionality of the absorption coefficient to the concentration of absorbing species, has been applied previously to dilute Fe-doped SrTiO_3_ single crystals by Maier et al. to study oxygen content spatially and temporally by optical means [21,22]. That work was based on earlier optical [38] and Mossbauer [39,40] studies relating different Fe valence states to different optical absorption signatures, with Fe^4+^ considered the main absorbing species near 2.8 eV (~443 nm). (More recent work has also related Fe valence states to optical absorption for the dilute case [41].) Assuming constant reflectivity vs. oxygen content, changes in transmitted light intensity (Δ*I*) can be related to changes in optical absorption coefficient (Δ*α*) – and therefore to changes in absorber concentration – in the material of thickness *L* under study:

(1) $$\Delta I=I_{0}e^{-\Delta \alpha L}$$

For films with thicknesses *L* well below the critical length (*l*_c_ = *D*/*k*), as in the present study [31], the optical absorption relaxation can be fit with the following equation for surface exchange-limited behavior:

(2) $$g\left(t\right)=\frac{\ln I\left(t\right)-\ln I_{i}}{\ln I_{f}-\ln I_{i}}=1-e^{-\frac{k_{\text{chem}}t}{L}}$$

where subscripts *i* and *f* refer to initial and final values of the relaxation, respectively, and *t* is time.

For the OTR measurements, the sample was held in a small (~1 cm diameter) quartz tube within a tube furnace, having windows to enable light transmission. A non-catalytic, and non-contaminating sample holder was used with minimal sample contact to keep the sample motionless during measurements. Monochromated (532 nm in the present work), collimated, and chopped light (Newport Apex Illuminator 70613NS with monochromator 74100, Irvine, CA, USA) was split prior to entering the chamber so that its incident intensity over time could be monitored by one photodetector. The remaining light was passed through the sample and measured by a second photodetector. The two photodetector and chopper signals were synchronized via lock-in amplifiers (Stanford Research Systems, Sunnyvale, CA, USA), and the ratio of transmitted to incident photodetector currents was employed in the analysis to remove contributions from any spurious light sources and their fluctuations. In the present work, data labeled ‘light transmission’ represent this ratio. The gas oxygen partial pressure was controlled by mixing high purity Ar and O_2_ via mass flow controllers (MKS Instruments, Andover, MA, USA) with a total flow rate of 100 sccm. The oxygen partial pressures were measured with the aid of a homemade Nernst-type yttria-stabilized zirconia sensor operated at a higher temperature in a separate tube furnace connected to the exhaust gas line from the OTR setup furnace. Because this second furnace used a large tube, its gas flush time was much longer than that of the OTR setup [18]. For this reason, time dependences of measured oxygen partial pressures shown later in the results section do not represent the exact time dependence of the gas environment in the OTR furnace. Gas flush time of a similar sized OTR tube with similar gas flow rate has been measured as <5 s using a residual gas analyzer [15]; however, that setup uses a 4-way switch for rapid gas changes, whereas the present setup relied upon electronic control of the mass flow controllers to switch mixtures.

Nearly simultaneous OTR and AC-impedance measurements of surface exchange kinetics were conducted on each of the two pieces of the same sample. In the first measurement run, using the first piece, a region of the film was coated with a sputtered porous Pt current collector [42], with a corresponding porous sputtered Pt counter electrode on the opposite side of the substrate. In the second measurement run, using the second piece, instead of Pt, a porous Au current collector was painted onto a portion of the film (Tanaka Kikinzoku International KK high purity Au paste, Tokyo, Japan). Pt wires fed through Al_2_O_3_ tubes were used to electrically connect the samples to the impedance analyzer. Light transmission (spot size approximately 2–3 mm) was measured through the remaining un-electroded portion of the samples. Impedance measurements were conducted using a Solartron 1260 impedance analyzer over the frequency range ~10^5^–$6\times 10^{-10}$ Hz with an applied ac voltage amplitude of 0.01 V. For certain measurements, a constant DC bias up to ±0.075 V was superimposed on the ac signal to modify the oxygen activity in the film by pumping oxygen in or out of the film through the ionically conducting substrate. While the oxygen activity is modified at the film/substrate interface, the thin film geometry enables rapid oxygen diffusion through the film compared to the slower film/gas interface oxygen exchange reaction, leading to reasonably homogeneous oxygen contents in the film. This approach, based on application of the Nernst relation (Equation (3)), provided a means for setting an ‘effective oxygen partial pressure’ (pO_2,eff_) in the film without changing the gas atmosphere (of partial pressure pO_2,gas_) in the tube furnace [43]:

(3) $${\text{pO}}_{2,\text{eff}}={\text{pO}}_{2,\text{gas}}\text{exp}\left(\frac{4F\Delta E}{\mathrm{RT}}\right)$$

where *F* is Faraday’s constant, Δ*E* is the applied DC bias across the substrate (after accounting for sources of voltage loss such as overpotentials), *R* is the gas constant, and *T* is temperature (in K).

As has been shown elsewhere [31], and as verified in the present study (see below), for half-cells in the present configuration containing dense, mixed conducting films with surface-exchange limited kinetics, the low frequency *R*–*C* circuit impedance arc in the impedance spectrum corresponds to the film response. The resistance (*R*) and capacitance (*C*) were determined by fitting the low frequency impedance arc to an equivalent circuit of a resistor in parallel with a constant phase element using ZView/ZPlot software (Scribner Associates Inc., Southern Pines, NC, USA). A chemical surface exchange coefficient can be estimated from the time constant (*τ* = R*C) of that arc [44]:

(4) $$k_{\text{chem}}=\frac{L}{\tau }$$

Additionally, the resistive contribution can be related to the electrical surface exchange coefficient [45]:

(5) $$k_{q}=\frac{k_{B}T}{4e^{2}\left(A\cdot R\right)c_{\text{O}}}$$

where *k*_B_ is the Boltzmann constant, *e* is the electronic charge, *A* is the electrode or current collector area, and *c*_O_ is the volumetric oxygen concentration. Note that *k*_*q*_ can be compared with *k*_chem_ by multiplication by a thermodynamic factor (*γ*):

(6) $$k_{\text{chem}}=k_{q}\cdot \gamma$$

It was therefore possible to estimate the thin film thermodynamic factors using Equation (6) by dividing the AC-IS derived *k*_chem_ values by the AC-IS derived *k*_*q*_ values [44]. It can also be seen, via comparison of Equations (4–6), that the film thermodynamic factor is inversely proportional to the film’s volumetric capacitance, which is largely a chemical capacitance but may be influenced by interfacial contributions. The two types of surface exchange coefficient result from different means of inducing oxygen surface exchange: chemical potential gradient vs. electrical potential gradient. Because of this fundamental difference and a desire to compare various measurements, some studies have undertaken to empirically and theoretically derive the relationship between *k*_*q*_ and *k*_chem_ [46,47]. The form of the thermodynamic factor in Equation (7) has previously been used for thin film mixed conductors [17] and therefore was also applied in the present work to provide another estimate of the value of the thermodynamic factor in the experimental conditions:

(7) $$\gamma =\frac{1}{2}\left(\frac{\partial \ln{\text{pO}}_{2}}{\partial \ln x_{\text{O}}}\right)$$

where *x*_O_ represents molar oxygen concentration (2.85 + *δ* for this material and its previously developed defect model; see Figure 6(a)). Data used in this calculation were taken from previous thermogravimetric analysis measurements of oxygen non-stoichiometry in bulk STF35 across a range of temperatures and oxygen partial pressures [48]. Bulk and thin film thermodynamic factors may fundamentally differ owing to variations in their point defect chemistry, as discussed in the introduction, so it is important to use the thin film values in this study to represent the point defect chemistry of the films under study. On the other hand, the experimental determination of thin film thermodynamic factors via the measured film capacitances is rendered challenging owing to the aforementioned possible interfacial contributions. Because of this potential limitation, the bulk thermodynamic factors were also applied in this work, as an estimate of the expected film values.

## 3. Results

**Figure 1.**
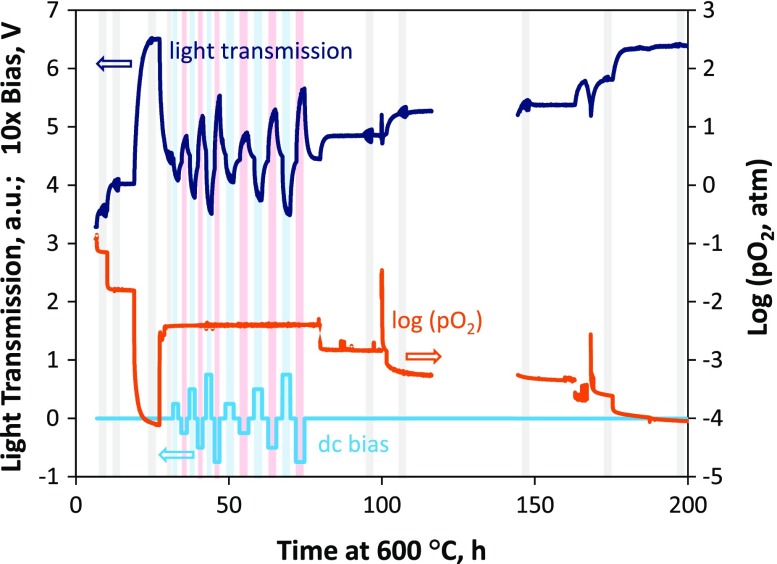
Overview of the raw data obtained during the first measurement run at 600 °C on the pre-aged STF35 film. Measured light transmission (dark blue, left axis) is shown to change over time in response to changes in both gas oxygen partial pressure (orange curve, right axis) and DC bias across the substrate (light blue curve, left axis). Shaded areas indicate times during which AC-impedance measurements were performed (blue shaded for positive DC bias and red shaded for negative DC bias).

In this section, results from the (nearly) simultaneous optical and AC-IS measurements performed on the same STF35 film at 600 °C are presented, using the porous Pt current collector except where noted. Prior to these measurements, the sample had undergone thermal treatment for a number of hours at temperatures up to 600 °C. The slower and more stable oxygen exchange kinetics of films that have been exposed to elevated temperatures over time [15,49] facilitated long-term measurements of *k*_chem_ at this elevated temperature by the optical technique. Without this prior annealing step, the kinetics would initially degrade quite rapidly [15,49], potentially distorting the measured oxygen partial pressure dependence of *k*_chem_. Figure 1 shows the raw data acquired during the measurements and also indicates, by the shaded gray areas, the times at which AC-impedance measurements were performed, with or without DC bias. The observed transmitted light intensity (ratio of the transmitted to incident photodetector current) is plotted as a function of the time during which the sample was maintained at 600 °C. Light transmission through the film changed as a result of modifications in its oxidized Fe (~Fe^4+^) concentration [14,21,38] in response to changes in the surrounding gas oxygen partial pressure and applied bias across the substrate. Increased light transmission corresponds to lower oxidized Fe concentrations, leading to a lower absorption coefficient [14]; such changes are observed in Figure 1 when the gas oxygen partial pressure is lowered or when a negative cathodic bias (negative on the STF side) is applied across the YSZ substrate. Note that, as mentioned in the experimental section, the oxygen partial pressure was measured in a larger, separate furnace with a much slower gas flush time than the OTR furnace, and therefore the gas change times shown in Figure 1 are much slower than those experienced by the sample (sample chamber gas flush times were discussed in the experimental section). In order to extract reliable *k* values from the relaxations, one can select experimental conditions that result in small changes in oxygen chemical potential and in constant thermodynamic factor during the relaxation [50]. The magnitudes of most of the oxygen partial pressure steps used in the OTR measurements are of a factor of 2–3, which is more appropriate for maintaining a relatively constant *k*_chem_ value during the relaxation; however, two steps early on in the measurements are larger. Fits of the OTR curves induced by these larger steps were included in the later analysis as they fit with the overall trend and also matched repeated measurements to these final oxygen partial pressures using smaller oxygen partial pressure steps. In other words, the determined *k* values were relatively insensitive to the step size. One can also see later in Figure 3 that the relaxation can be modeled well with linear surface exchange-limited behavior with a single *k* value and in Figure 6 that the thermodynamic factor is not changing significantly with small steps in oxygen partial pressure.

**Figure 2.**
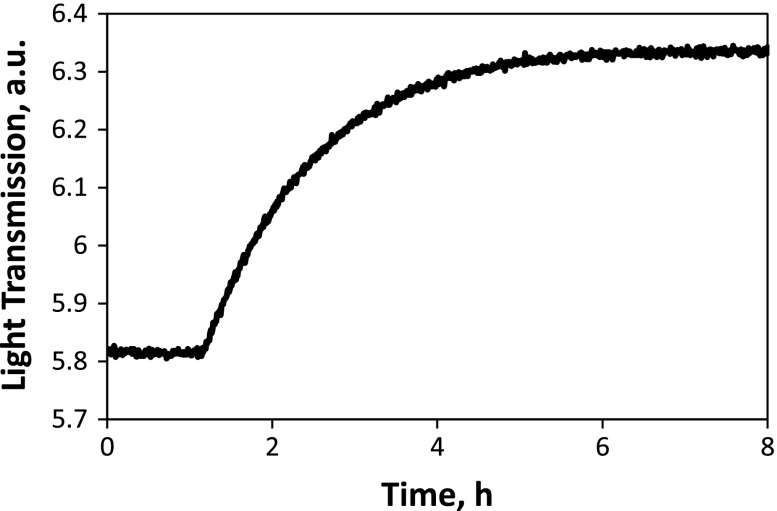
Example of OTR curve upon lowering oxygen partial pressure from 2.5 × 10^−4^ to 1.1 × 10^−4^ atm at 600 °C for the aged STF35 film.

**Figure 3.**
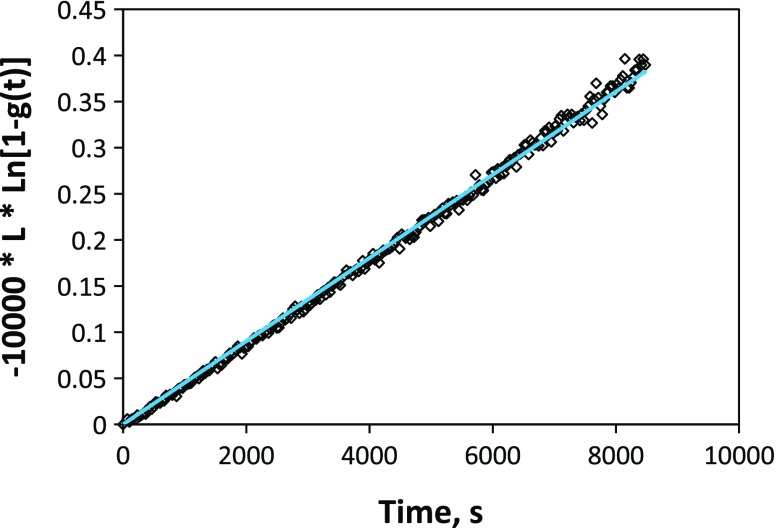
Fit by Equation (2) for surface exchange-limited kinetics, demonstrating the expected linear behavior and that was used to extract *k*_chem_ from the slope. Film thickness was ~200 nm.

An enlarged OTR curve obtained during the measurement, for a pO_2_ change of 2.5 × 10^−4^ to 1.1 × 10^−4^ atm, is given in Figure 2. The transmitted light intensity increases as the sample reduces in response to the drop in oxygen partial pressure. In this case, owing to the low oxygen partial pressure and prior aging of the sample, the response time for the film to equilibrate is quite slow – several hours. The kinetics of equilibration do appear to be surface exchange-limited (as expected for STF35 at this temperature and with thickness well below the critical length), as supported by Figure 3, where an excellent linear fit by a rearrangement of Equation (2) is observed. Here, the slope corresponds to 10000*k_chem_.

**Figure 4.**
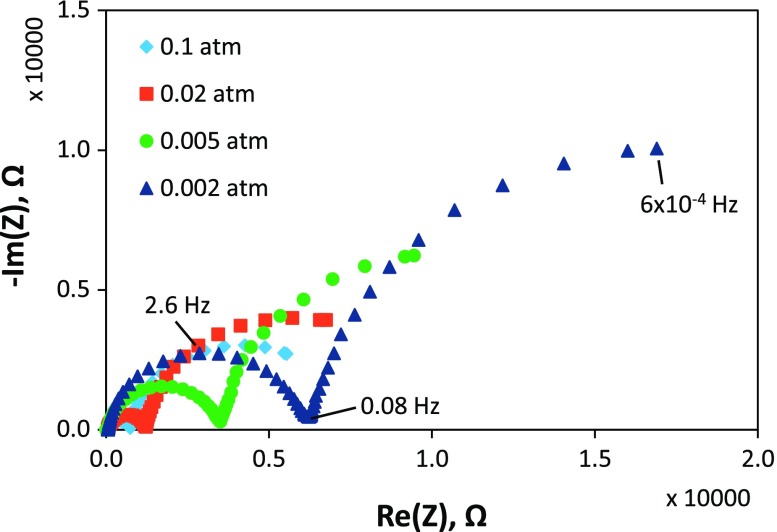
AC impedance spectra measured on the porous Pt/YSZ/STF/porous Pt cell at 600 °C in different oxygen partial pressures, as shown in the legend. Selected frequencies are indicated on some points for the spectrum measured in 0.002 atm O_2_.

**Figure 5.**
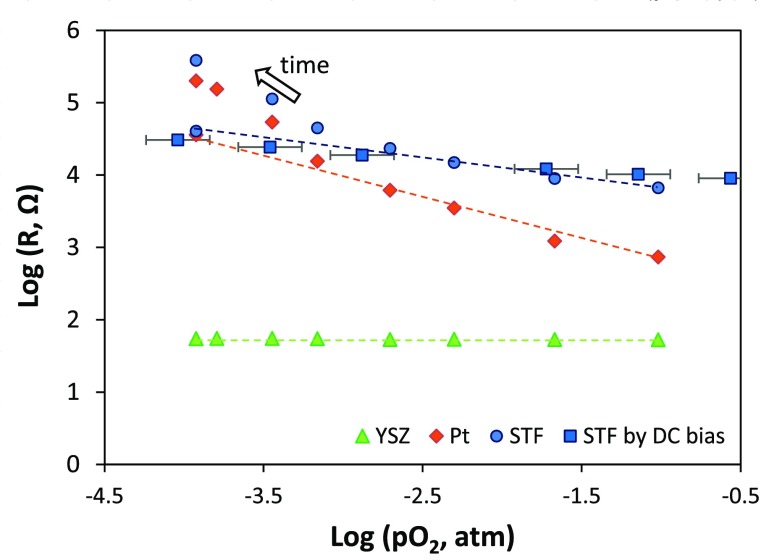
Oxygen partial pressure dependence of high frequency (YSZ), medium frequency (Pt counter electrode) and low frequency (STF) resistances, from equivalent circuit fitting of the impedance spectra, for the porous Pt/YSZ/STF/porous Pt cell at 600 °C. Dashed lines indicate guides to the eye showing the slopes for initial data prior to more significant degradation (~0 for YSZ, −0.61 for Pt, and −0.28 for STF). Increasing deviation from these slopes were observed for the Pt and STF contributions after increasingly long times (>100 h).

Examples of impedance spectra obtained in different gas atmospheres (at selected times corresponding to shaded gray areas in Figure 1) are shown in Figure 4, for the first measurement run with the porous Pt current collector and counter electrode. The oxygen partial pressure for each measurement is given in the legend, and measurement frequencies are indicated for selected points. It was not possible to measure the full low frequency arc because measurements down to even lower frequencies would have taken prohibitively long periods of time; however, sufficient points are available for determining its resistance by equivalent circuit fitting. It can be seen that, as the oxygen partial pressure was lowered, the size of the medium and low frequency impedance arcs increased. There is an additional high frequency resistive feature that is not visible at this scale on the plot, such that the whole spectrum can be modeled as a R(RQ)(RQ) equivalent circuit (in Boukamp notation [51]), i.e. a resistor in series with a parallel resistor-constant phase element in series with another parallel resistor-constant phase element. The constant phase elements fit to the equivalent circuit exhibited behavior close to that of perfect capacitors, with fitting exponents of ~0.9 (little arc depression from a semicircle). As mentioned in the experimental section, for samples of this geometry and materials properties, the low frequency arc typically corresponds to the electrode film (STF) response [31]. However, porous Pt counter electrodes and current collectors had not been applied to STF cells previously to our knowledge, so it was important to verify the origins of each impedance feature. Features may be allocated primarily by their capacitances along with their temperature- and oxygen partial pressure-dependences; for example, the oxygen partial pressure dependence of their resistances is shown in Figure 5. Here, the high frequency feature was attributed to YSZ as it exhibited a pO_2_-independent resistance and an activation energy close to 1 eV, ascertained by separate temperature-dependent measurements, as expected for YSZ [52]. The low frequency feature was attributed to STF on the basis of its large capacitance, which is typical of the chemical capacitance for STF as reported previously [31], as well as the pO_2_-dependence of its resistance and corresponding *k*_*q*_, which approximately matched that of *k*_chem_ measured optically. The remaining intermediate frequency feature was attributed to the Pt counter electrode as its resistance exhibited a steeper dependence on pO_2_. In impedance measurements of similar cells, the intermediate frequency feature has also been attributed to counter electrodes, e.g. porous Ag paste [31]. Deviation from the initial pO_2_-dependence was observed for the STF and Pt resistances after long measurement times, as shown in Figure 5; therefore, the guides for the eye include only the points measured at early times, prior to significant degradation. For this porous Pt/YSZ/STF/porous Pt cell, the log(*R*) vs. log pO_2_ slopes were ~0, −0.61, and −0.28, for YSZ, Pt counter electrode, and STF, respectively. For the case of the porous Au current collector, in measurement run 2 (not shown) the initial STF slope prior to more significant degradation was −0.30 for the pO_2_ range 0.21–0.003 atm.

**Figure 6.**
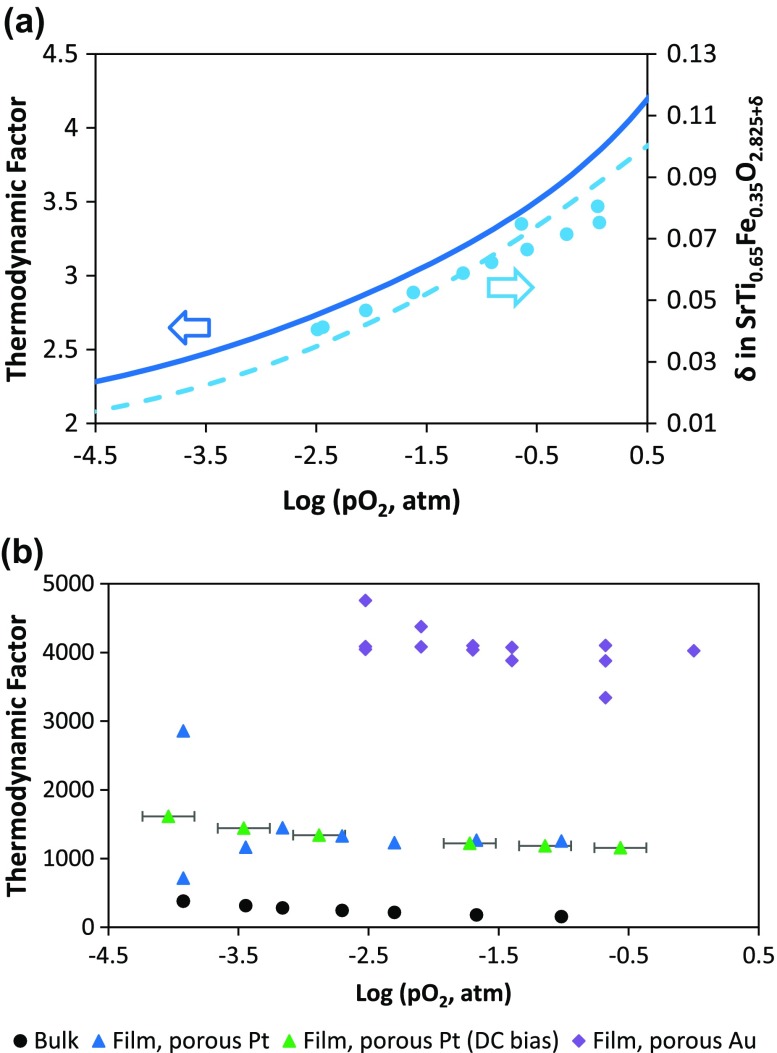
(a) Calculated bulk thermodynamic factor as expressed in Equation (7), from thermogravimetric oxygen non-stoichiometry data, also shown with fit to a broader range of data, from Ref. [48]. (b) Estimated thin film thermodynamic factors from the ratio of AC-IS derived *k*_chem_ to *k*_*q*_ (or from the measured capacitance), as in Equations (4–6), for different current collectors.

Figure 6(a) shows the estimated bulk thermodynamic factor calculated using Equation (6) at 600 °C as a function of oxygen partial pressure alongside the corresponding bulk oxygen non-stoichiometry measurements (Ref. [48]) and fit (Ref. [48]). The oxygen non-stoichiometry fit represents the best fit to data over a wide range of temperatures and oxygen partial pressures using the model in Ref. [48], although it is not a perfect fit to the small selection of data shown here. The bulk thermodynamic factor is calculated to vary depending on oxygen partial pressure (shown) and temperature (not shown), although the pO_2_ dependence is relatively mild – the thermodynamic factor changes by a factor of ~3–4 for a change in pO_2_ of 5 orders of magnitude. Figure 6(b) compares the thin film thermodynamic factors, estimated from the ratio of *k*_chem_ to *k*_*q*_ from impedance measurements, to the bulk thermodynamic factor for the same conditions. Some scatter in the thin film data likely originates from the ongoing degradation during the measurements. One can observe that the thin film values are higher than the bulk values, though the pO_2_ dependence is similarly shallow. Different current collectors yielded different thermodynamic factors. These large differences suggest a possible role of interfacial capacitances contributing to the measured STF capacitance. An interfacial capacitance would be in series with the STF chemical capacitance, thereby capable of lowering the effective measured capacitance and increasing the associated thermodynamic factor.

**Figure 7.**
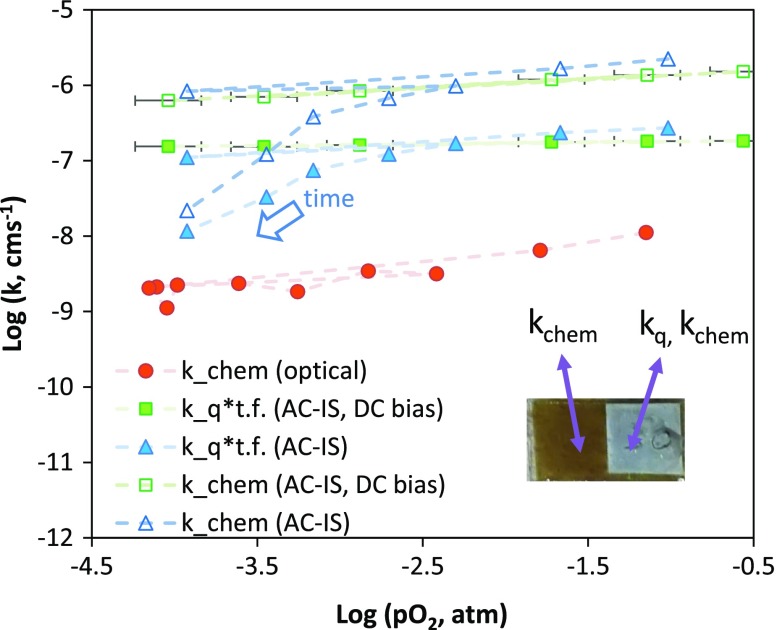
Comparison of (nearly) simultaneously measured *k*_chem_ and *k*_*q*_ values for STF35 at 600 °C (t.f. stands for bulk thermodynamic factor = *γ*) using a sputtered porous Pt current collector on a portion of the film. Inset is a photo of the sample for simultaneous OTR of the native film and AC-IS measurements using the porous Pt current collector.

Figure 7 then compares the optically measured *k*_chem_ values at 600 °C as a function of oxygen partial pressure on an aged sample with *k*_*q*_ multiplied by the bulk thermodynamic factor and *k*_chem_ measured on the same sample during the same course of measurements by AC-IS. This figure is for the cell with the porous Pt current collector. As described in the experimental section, the reported AC-IS measurements were performed in two ways: one as a function of gas oxygen partial pressure and the other as a function of DC bias applied across the ionically conducting YSZ substrate to create an effective oxygen activity in the samples while keeping the external gas atmosphere constant at pO_2, gas_ = 0.005 atm. In this case the x-axis represents ‘effective oxygen partial pressure’ obtained by the Nernst equation (Equation (3)) [43]. For the OTR derived *k*_chem_ values, the pO_2_ represents the final pO_2_ of the relaxation. Lines connect data points, with the arrow showing the time progression and order in which measurements were conducted, beginning in 0.21 atm O_2_. One initially observes a similar, approximately ${\text{pO}}_{2}^{1/4}$ dependence for the optical (*k*_chem_) and impedance (*k*_chem_ and *k*_*q*_) measurements. The slope is slightly shallower for the bias-dependent AC-IS measurements compared to the gas partial pressure-dependent AC-IS measurements. Over time, a significant decrease of the AC-IS derived *k* values is observed, deviating from the initial oxygen partial pressure dependence. A similar magnitude of decline in the optically derived *k*_chem_ over that time period is not observed. The AC-IS derived values (*k*_q_*bulk *γ* and *k*_chem_) are several orders of magnitude larger than those obtained from OTR (*k*_chem_). The difference in bulk vs. apparent thin film thermodynamic factors is evident in the difference between AC-IS derived *k*_q_*bulk *γ* and AC-IS derived *k*_chem_, which is equal to *k*_q_*thin film *γ*.

**Figure 8.**
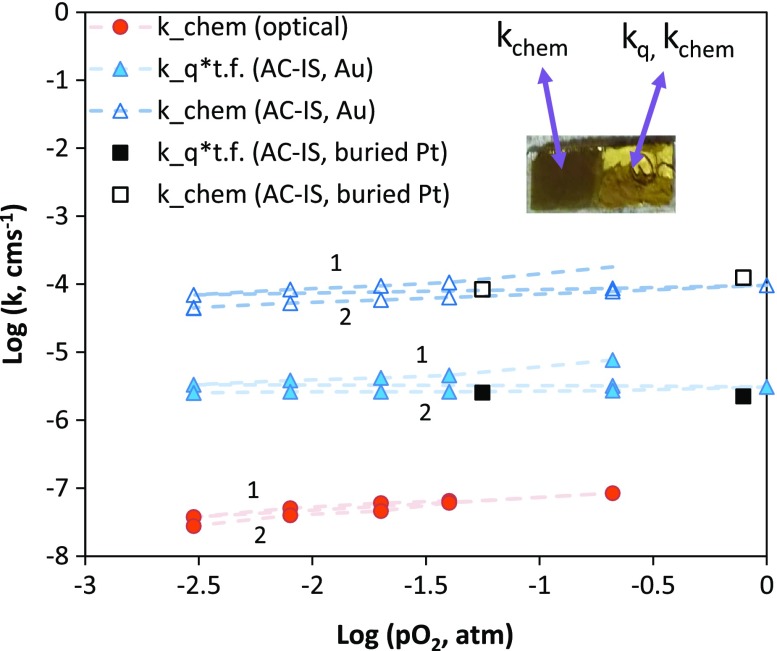
Comparison of (nearly) simultaneously measured *k*_chem_ and *k*_*q*_ values for STF35 at 600 °C (t.f. stands for bulk thermodynamic factor = *γ*) using a painted porous Au current collector on a portion of the film. Sample is another piece of the sample used in Figure 7. Inset is a photo of this piece used for simultaneous OTR of the native film and AC-IS measurements with the porous Au current collector. Approximate data for buried Pt finger current collectors are also included.

In order to discover whether similar differences between impedance and optical *k* values would be observed with other current collectors, a second set of measurements was performed using a painted porous Au current collector on top of the STF film instead of Pt. The sample was another piece of the same film used with the porous Pt current collector in the previous results. The results, shown in Figure 8, reveal a similar outcome with Au as for the case of the porous Pt current collector, namely that the impedance derived *k* values are orders of magnitude higher than the optically derived *k* values. In this case, all the *k* values are shifted higher relative to Figure 7, because the sample had not been annealed for as long (to cause aging) prior to the measurements. In general *k* values may vary orders of magnitude between STF samples of nominally similar compositions but with different thermal histories and presumably different surface chemistries [15]; for this reason, meaningful comparison of *k* values between different measurement runs or samples is difficult to achieve. In this case the optical *k*_chem_ value at 0.21 atm O_2_ is in reasonable agreement with a *k*_chem_ of approximately 5 × 10^−7^ cm/s for STF50 extrapolated from reported higher-temperature electrical conductivity relaxation results [29] to this temperature, while the impedance *k*_*q*_ values are in better agreement with a tracer k* value [28] for STF50 of approximately 6.3 × 10^−7^ cm/s at this temperature and a slightly higher oxygen activity (aO_2_ = 0.5). Measurements were performed from high to low pO_2_ and then repeated from high to low pO_2_ again (sequences labeled 1 and 2 in Figure 8). A small amount of degradation was observed in the decrease of *k* values by the second sequence and the transition to shallower pO_2_-dependence over time for *k*_q_*bulk *γ*, but the degradation again appears larger for the impedance derived data than for the optical results. Figure 8 also includes limited AC-IS derived approximate *k* values for a third case using sputtered buried Pt fingers as a current collector on a different but similar thickness (212 nm vs. 200 nm) STF film (Pt between the STF and the YSZ rather than at the STF-gas interface). Despite the possible inadequate current collection of the fingers, relatively high *k* values are obtained from these impedance measurements, as with the painted electrodes. Additionally, one observes a similar ratio of *k*_chem_ to *k*_*q*_ for the buried electrode impedance measurements; in other words, the volumetric capacitance and estimated thin film thermodynamic factor are the same for the case of the painted, porous Au electrode and the buried Pt finger electrode.

## 4. Discussion

In terms of magnitude, there was poor agreement between the optically derived surface exchange coefficients (*k*_chem_) and those measured by AC-IS (*k*_chem_ and *k*_q_*γ), even when different current collectors were used. The OTR technique measures surface exchange coefficients in a continuous and *in situ* manner, without requiring any contacts to the sample. Few techniques offer this combination of possibilities, important for evaluating dynamic processes under *in situ* conditions. On the other hand, the AC-IS technique (as well as electrical conductivity relaxation) requires metallic current collectors that typically are interfacing with the exact surface of samples being probed, unless they are buried; this means that in theory the current collectors could markedly modify the measured *k* values, via enhanced catalytic activity, physical blocking of active surfaces, modification of space charge regions [53] and/or poisoning impurities (e.g. current collectors could act as sources or sinks for surface-poisoning species such as Cr, S, Si, Sr), etc. Additionally, oxygen exchange is induced by different means in the two cases (chemical potential gradient vs. electrical potential gradient as the driving force), although for small driving forces this factor alone should not contribute to different *k* values [28]. Further, electronic charge transfer, required for electrochemical oxygen exchange, may occur more readily at metal current collectors relative to the films, which have limited electronic conductivity. One or more of these effects may explain the differences observed in optical vs. impedance results. A similar enhancement of AC-IS derived *k*_q_*γ relative to OTR derived *k*_chem_ was previously observed in the case of Ce_0.9_Pr_0.1_O_2-x_ thin film electrodes using sputtered porous Pt current collectors, studied under similar oxidizing conditions, and in that case the difference was hypothesized to be caused by a possible catalytic contribution from Pt, elevating the *k*_*q*_ value [17]. In further studies of Ce_0.9_Pr_0.1_O_2-x_ thin film electrodes, based on the different pO_2_-dependences of *k*_*q*_ and *k*_chem_, it was hypothesized that Pt might change the rate-limiting step and/or reaction site: the reaction, when Pt is present, may take place at the film/Pt/gas triple phase boundary rather than over the full surface of the film, at the film/gas interface [42]. One potential follow-up study could evaluate in more detail than in Figure 8 the response when buried current collectors are employed, since these would not modify the film/gas interface where oxygen exchange takes place. However, buried current collectors can introduce other complications. STF films grow with different orientations and microstructures on Pt vs. on YSZ, so the film being probed optically would not be exactly the same as the film being probed by impedance in that case. Buried electrodes can introduce shadowing effects during growth of films on top, which can cause problems for film continuity for quite thin films as in the present case. Furthermore, given the high in-plane resistance of the films, adequate current collection from buried electrodes likely can only be achieved by lithographic patterning with micron-scale spacing.

The OTR derived *k*_chem_ and initial AC-IS derived *k*_chem_ and *k*_*q*_ values exhibited a similar oxygen partial pressure dependence, but differences appeared after long annealing times. While the *k*_chem_ values did not exhibit very significant degradation over the course of these measurements (particularly for the measurements with the Pt current collector, where the STF was already significantly ‘aged’ prior to measurements), the AC-IS derived *k* values became systematically lower after long measurement times (particularly > 100 h), deviating from the early behavior. In the case of the cell with the porous Pt current collector, from the results of the impedance fitting (Figure 5) it is apparent that both the Pt counter electrode and STF-Pt current collector resistances (but not the YSZ resistance) began to increase by similar factors around the same time. Therefore, it appears likely that some chemical/morphological changes in the Pt current collector were primarily responsible for the longer term degradation observed in the STF AC-IS derived *k* values. A similar effect may be present for the case of the Au current collector since the AC-IS derived *k* values demonstrated more degradation than the OTR derived values, albeit over a short measurement time frame. Here it can be seen that a particular benefit of simultaneous OTR and AC-IS measurements is the ability to separate the impact of native STF ‘aging’ (surface chemical changes) from that of current collector-induced ‘aging.’ More generally, it can be noted that, since the surfaces of many electrode materials are chemically dynamic, leading to variations in *k* over time (at least in the initial ~ 40 h of measuring [15,49]), the present approach underlines the advantage of simultaneous comparison of different methodologies, wherein the same sample is probed with the exact same thermal and environmental history to minimize variability in surface chemistry that could otherwise contribute to different *k* values when evaluated by separate measurements on separate samples.

Finally, within the impedance derived *k* values, reasonable agreement was observed between the pO_2_ dependence obtained by changing the gas oxygen partial pressure and the ‘effective pO_2_’ dependence by changing the DC bias applied across the YSZ substrate. The magnitudes of *k* values for gas vs. bias control were similar, and the slopes of log *k* vs. log pO_2_ were also similar, within uncertainty/error. Slight differences in the magnitudes and trends could be due to a few effects. First, while both techniques can set an equal oxygen activity in the film, the amount of oxygen present in the gas phase to exchange with the sample is different in the two cases. If the rate-limiting step for oxygen surface exchange is dominated by point defect concentration(s) then the two approaches should produce similar *k* results. If the rate-limiting step is influenced by oxygen availability in the gas phase (possibly oxygen adsorption/desorption) then the two approaches would give different *k* results. Second, there is a finite equilibration time to reach the new stoichiometry after applying the DC bias; however, the impedance measurement began immediately. This observation suggests a possible offset in point defect density during the initial impedance measurement vs. the steady-state point defect chemistry that would be achieved after applying the DC bias for longer times. To check for this effect, impedance measurements were performed with different DC bias application times; no significant differences in STF resistance (or *k*) values were, however, observed, suggesting that the time taken to reach steady-state oxygen concentrations in the region of the current collectors was faster than the time it took the impedance scan to reach the STF response’s frequency range. Note that the apparent long equilibration times after applying a DC bias observed optically in Figure 1 were measured on the other side of the sample, away from the application of the bias; these equilibration times may have been limited by the lateral (in-plane) diffusion of oxygen in the STF film (with a possible additional influence from oxygen surface exchange) and may be different to the equilibration times near the current collector. Third, no reference electrode was used in the present work that could have removed overpotential contributions to voltage losses when DC bias was applied. In the present configuration, various losses, including ohmic losses in the substrate and overpotentials at the electrodes, could have resulted in a lower effective pO_2_ change than that calculated by the Nernst equation using the full applied voltage. The ohmic losses were observed to be small, with low currents. To check if there was a significant difference between the oxygen activity achieved by gas oxygen partial pressure control vs. DC bias application, resulting from overpotentials, the stoichiometry of the film vs. DC bias was compared with the stoichiometry as a function of gas partial pressure, using the measured optical transmittance intensity (not shown) and the chemical capacitance/ thermodynamic factor (Figure 6(b)) as indicators of stoichiometry. This comparison suggested that the Nernst equation (Equation (3)) using the full applied DC bias gave a reasonable approximation of the effective oxygen partial pressure within the film. With increasing measurement temperature the magnitude of the electrode overpotentials decreases, so a reference electrode becomes ever more important as the measurement temperature decreases.

## 5. Conclusions

Techniques that can evaluate point defect equilibria and kinetics in oxide thin films *in situ* are needed, in order to better understand and rationally tailor film performance for various applications. In the present work, the OTR technique was applied to thin films of the mixed conductor SrTi_0.65_Fe_0.35_O_3-x_, with a focus on evaluating its oxygen surface exchange kinetics at 600 °C as a function of oxygen partial pressure. At the same time, AC-IS measurements were performed on the same sample in order to compare the assessment of oxygen exchange kinetics by the two techniques under nearly identical thermal and environmental conditions. *k*_chem_ values, measured optically, exhibited a similar oxygen partial pressure dependence to the initial *k*_chem_ and *k*_*q*_ values measured by impedance spectroscopy; however, they were orders of magnitude lower than the impedance derived *k*_*q*_ values normalized (multiplied) by the thermodynamic factor (calculated on the basis of previously measured bulk non-stoichiometry and additionally estimated for the films on the basis of their measured capacitances). The results suggested that the both the sputtered porous Pt and painted porous Au current collectors used for impedance measurements contributed to enhancing the impedance derived *k*_chem_ and *k*_*q*_. Additionally, significant long-term degradation of the apparent *k* values measured by impedance spectroscopy was observed, which was attributed to chemical or morphological changes in the current collector. Such degradation was understandably absent from the optically derived *k*_chem_ values. OTR therefore offers a means of evaluating thin film oxygen surface exchange kinetics continuously, under realistic operating conditions. Unlike the impedance approach, the optical method additionally enables measurement of *native* oxide surface behavior without complications arising from the application of metallic current collectors to the surface.

## Disclosure statement

No potential conflict of interest was reported by the authors.

## Funding

This work was supported by the JSPS Kakenhi Grant-in-Aid for Young Scientists (B) [project number JP15K18213] (to N.H. Perry) and by the International Institute for Carbon-Neutral Energy Research (WPI-I^2^CNER, MEXT) at Kyushu University, Japan. J.J. Kim and H.L. Tuller thank US-DOE Basic Energy Sciences [grant number DE-SC0002633] for financial support.
